# Need‐Based Mental Health Aid Allocation to Disadvantaged Patients Toward Universal Health Coverage in Bangladesh

**DOI:** 10.1002/puh2.70197

**Published:** 2026-03-13

**Authors:** Saiful Islam Saif, Sultana Nasrin, Sayedul Ashraf Kushal, Yahia Md Amin, Woarisha Alam, Md. Tajul Islam

**Affiliations:** ^1^ Department of Research & Development LifeSpring Well‐Being Foundation Dhaka Bangladesh; ^2^ Department of Statistics Lund University Lund Sweden; ^3^ Department of Finance & Admin Spanish Embassy of Bangladesh Dhaka Bangladesh; ^4^ Department of Public Health North South University Dhaka Bangladesh

**Keywords:** need‐based mental‐health aid, out‐of‐pocket costs, socioeconomic equity, universal health coverage

## Abstract

**Introduction:**

Out‐of‐pocket (OOP) healthcare costs remain prohibitive in low‐resource settings, often pushing families into poverty. Universal health coverage (UHC) goals emphasise protecting households from financial hardship due to medical expenses.

**Objectives:**

This study examines which socioeconomic and demographic factors predict the amount of free mental healthcare support received by applicants.

**Methodology:**

We conducted a cross‐sectional analysis. Descriptive statistics summarised 286 applicant profiles (e.g., 66.8% female and 49% aged 28–48). Spearman and Pearson correlations assessed the relationship between applicants’ monthly treatment‐related OOP costs and aid received (log‐transformed values). A multiple linear regression was then fitted with log (free care amount) as the outcome and predictors including age group, sex, occupation, marital status, income source, reason for support and log (monthly treatment‐related OOP cost).

**Results:**

The applicant profile was predominantly young, unmarried, female students relying on parental support, seeking aid mainly for financial hardship. Spearman's *ρ* showed a small but significant positive correlation between treatment cost and aid amount, confirmed by Pearson's *r* after log transformation (*r *≈ 0.17, *p* < 0.01). In regression analysis, higher treatment cost strongly predicted more free aid (*β* ≈ 0.18, *p* = 0.003), indicating that donors allocate more resources to costlier cases. Households supported by a spouse's income received significantly less aid (*β* ≈ −0.93, *p* = 0.038), suggesting that more stable households needed less assistance. Parental income support was marginally associated with reduced aid (*p* ≈ 0.07), whereas student status showed a borderline positive effect (*p* ≈ 0.08).

**Conclusion:**

This study highlights that free mental healthcare support in a resource‐poor setting is driven primarily by treatment cost and applicants’ perceived need. Those with higher medical expenses receive more aid, aligning with the principle of need‐based assistance.

## Graphical abstract

De‐identified administrative records of 286 mental health aid applicants in Bangladesh are analyzed to examine need‐based aid allocation. Higher out‐of‐pocket treatment costs are associated with larger aid awards. Applicants with spouses as primary earners receive substantially less aid, while students or those without stable income receive slightly more. Financial hardship appears to influence aid allocation more strongly than stated mental health need.

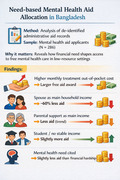

## 1 Introduction

Out‐of‐pocket (OOP) payments for medical care can create severe financial hardship for low‐income households. Research shows that in many low‐ and middle‐income countries, large OOP health expenses push people into poverty and discourage them from seeking care [1, 2]. The World Health Organization (WHO) reports that by 2019, nearly one billion people worldwide were subjected to catastrophic health spending, with approximately 344 million driven into extreme poverty by medical costs [3, 4]. Achieving universal health coverage (UHC) requires reducing such financial barriers. In settings lacking comprehensive insurance, free healthcare schemes or charitable aid are critical safety nets for underserved populations [5, 6].

Socioeconomic and demographic factors strongly shape health and healthcare access. The WHO emphasises a ‘social gradient’ in health: Individuals with lower socioeconomic position (income, education and occupation) tend to experience worse health and face greater barriers to care [7]. Numerous studies confirm that wealthier, more educated and urban households use health services more frequently than the poor. For example, Khanam and Hasan found in Bangladesh that children from higher wealth families and with educated parents were significantly more likely to receive treatment for illnesses [8].

However, evidence is mixed on how such factors influence access to free or subsidised services specifically. Free‐care policies (e.g., fee waivers for maternal/child health) have been shown to reduce inequities [9]. Samadoulougou et al. report that introducing free healthcare in Burkina Faso substantially diminished pro‐rich disparities in paediatric fever treatment [10]. Nonetheless, the actual distribution of individual‐level free aid (such as hospital or NGO charity funds) may still reflect biases. Factors like marital status, employment or income sources could affect perceived need [11, 12]. For instance, Ayanore et al. found that Ghanaian adults who lost family financial support during COVID‐19 were much less likely to obtain care, highlighting the role of economic security [13].

These issues are particularly silent for mental healthcare. Mental health conditions are a leading cause of disability worldwide and are strongly associated with sustained OOP expenditures due to long‐term treatment needs and limited public financing, particularly in low‐ and middle‐income countries [14, 15]. High treatment costs can lead to delayed care, treatment discontinuation and worsening mental health outcomes, disproportionately affecting socioeconomically vulnerable individuals [16]. Unlike many physical illnesses, mental health needs are often compounded by stigma, reduced earning capacity and weaker household support, which may influence both access to care and the allocation of financial assistance [17].

The WHO estimates that depression and anxiety disorders alone cost the global economy approximately $1 trillion annually in lost productivity, yet less than 2% of health budgets in low‐income countries are allocated to mental healthcare [15, 18]. In Bangladesh, where the mental health treatment gap exceeds 90% and psychiatrist density remains extremely low (approximately 0.44 per 100,000 population), OOP payments for psychiatric consultations often range from 2000 to 5000 Bangladeshi Taka per session, and psychotropic medications represent catastrophic expenses for most households [19, 20, 21]. These financial barriers exacerbate clinical suffering and reinforce cycles of untreated illness and economic decline. Despite this, empirical evidence on how socioeconomic factors shape the distribution of free mental health aid remains limited.

In this context, the LifeSpring Well‐being Foundation's Mental Health Aid Program provides a relevant case study, offering need‐based financial support by covering consultation and counselling costs for individuals facing financial hardship following a brief eligibility assessment. By reducing OOP payments for the duration of treatment, the program operationalises financial risk protection and contributes to UHC in a resource‐constrained mental health system. This study investigates the socioeconomic predictors of the amount of free mental healthcare aid received by applicants in such a setting. By analysing data from 286 applicants to a mental health aid program (supported by LifeSpring Well‐being Foundation), we seek to identify which individual characteristics are associated with larger or smaller aid allocations. Better knowledge of these determinants can inform more equitable targeting of scarce philanthropic and public health resources.

## 2 Methodology

### 2.1 Sample and Data Collection

We analysed administrative records from 286 individuals who applied for free mental healthcare support at a LifeSpring Well‐being Foundation‐affiliated mental health aid program. All applicants had documented monthly treatment‐related OOP costs and socio‐demographic information. Two applicants had missing data on the aid amount and were excluded, yielding a final sample of 284 for regression analysis.

The study was reviewed by LifeSpring Consultancy Limited IRB and determined to be exempt from formal ethics approval due to use of existing, de‐identified administrative data (Reference No. 2023/LS/IRB/01). The IRB confirmed that all personal identifiers were removed prior to analysis, thereby negating the need for ethical approval. Written informed consent for the use of data was obtained from participants at the time of enrolment in the aid program, and no additional consent was required for this retrospective analysis. All identifying information was removed before analysis, the data were stored securely, and only aggregated results are reported to ensure participant anonymity. Administrative records collected between 2nd October 2023 and 7th January 2025 were used for this study.

### 2.2 Variables

The dependent variable was the total value of free care provided to each applicant (in Bangladeshi taka). Because aid amounts and costs were skewed, we log‐transformed continuous variables for analysis. Key independent variables included monthly treatment‐related OOP cost, applicant's total medical expenses for the current condition and log‐transformed. We also grouped applicants by age (7–27, 28–48, 49–69 and 70–90 years) to capture life‐stage differences, sex (male or female) and occupation (student, jobless or employed). To see where their money comes from, we noted whether their main household income was provided by parents, spouse, themselves, relatives or someone else. We recorded marital status as unmarried, married, divorced or widowed, and finally, we classified the stated reason for seeking aid as either financial hardship, mental‐health needs or something else.

Information was self‐reported by applicants during intake interviews and verified against available billing records or prescription receipts when provided. Applicants were asked to document: (i) consultation fees paid to mental health professionals, (ii) medication costs and (iii) transportation expenses for treatment visits for the preceding month. Program staff conducted brief validation checks for consistency (e.g., cross‐referencing stated consultation frequency with typical market rates), but comprehensive auditing was not feasible.

### 2.3 Statistical Analysis

We first computed descriptive frequencies to profile the applicants. Bivariate associations between monthly treatment‐related OOP costs and free aid were assessed using Spearman's rho and Pearson's correlation on the logged values. Next, we fitted a multiple linear regression model with log (aid amount) as the outcome and the above predictors as independent variables. This model tested which factors were significantly associated with larger or smaller aid amounts, controlling for others. All analyses were performed in R (v4.1.2).

The linear regression model with log‐transformed outcome and cost variables was chosen primarily to address the strong right‐skewness observed in both the free‐care amount and monthly cost variables. Log transformation improved the approximate normality of residuals, reduced heteroscedasticity and allowed regression coefficients to be interpreted as proportional (percentage‐based) effects, which is meaningful in the context of cost‐related outcomes. We acknowledge that alternative modelling strategies, such as generalised linear models with a gamma distribution and log link, could also be appropriate for skewed cost data. However, given the exploratory nature of this study, the moderate sample size and the interpretability of the log‐linear framework, we opted for the linear regression approach as a transparent and commonly used method in health‐economic analyses.

### 2.4 Program Context

LifeSpring Well‐being Foundation is the charitable wing of LifeSpring Consultancy Limited, a non‐profit mental healthcare service provider in Bangladesh. The Foundation aims to increase access to mental healthcare and improve the overall well‐being of community members, especially those who are marginalised or underserved. The Foundation currently provides humanitarian assistance to coastal populations affected by climate change, mental health aid services for disadvantaged people, conducts research on mental health and public health issues and operates an online suicide helpline to assist emotionally vulnerable people.

The LifeSpring Well‐being Foundation's Mental Health Aid Program enables disadvantaged individuals to apply online or on‐site. After a brief eligibility check confirming financial hardship, the Foundation covers every consultation and counselling fee for as long as treatment is required, directly advancing UHC by ensuring quality, need‐based mental healthcare for those who otherwise could not afford it. The program started on 2nd October 2023 and continues to date.

## 3 Results

Table 1 shows the sample characteristics. The median applicant age fell in the 28–48 year group (49%), and 66.8% were female. A majority (61.5%) were students, 25.9% were unemployed and only 12.6% had regular employment. Over half (56.6%) reported their parents as their income source, with only 14.3% self‐supporting; 18.7% relied on a spouse or relatives (Table 1). Most applicants (62.2%) were unmarried. Financial hardship was the most common stated reason for aid (48.6%), followed by mental health issues (34.6%). In summary, the typical applicant was a young, unmarried female student living with parents and seeking aid for financial need.

**TABLE 1:** Demographic characteristics of applicants.

Variable	Category	Frequency (%)
Age group	7–27 years	26 (9.1)
	28–48 years	139 (48.6)
	49–69 years	92 (32.1)
	70–90 years	29 (10.1)
Sex	Female	191 (66.8)
	Male	95 (33.2)
Occupation group	Job	36 (12.7)
	Jobless	74 (25.9)
	Student	176 (61.5)
Income source	Others	2 (0.7)
	Parents	162 (56.6)
	Relative	27 (9.4)
	Self	41 (14.3)
	Spouse	54 (18.9)
Marital status	Divorced	9 (3.1)
	Married	95 (33.2)
	Unmarried	178 (62.2)
	Widowed	4 (1.4)
Reason for support	Financial hardship	139 (48.6)
	Mental health issues	99 (34.6)
	Other	48 (16.8)

**FIGURE 1:**
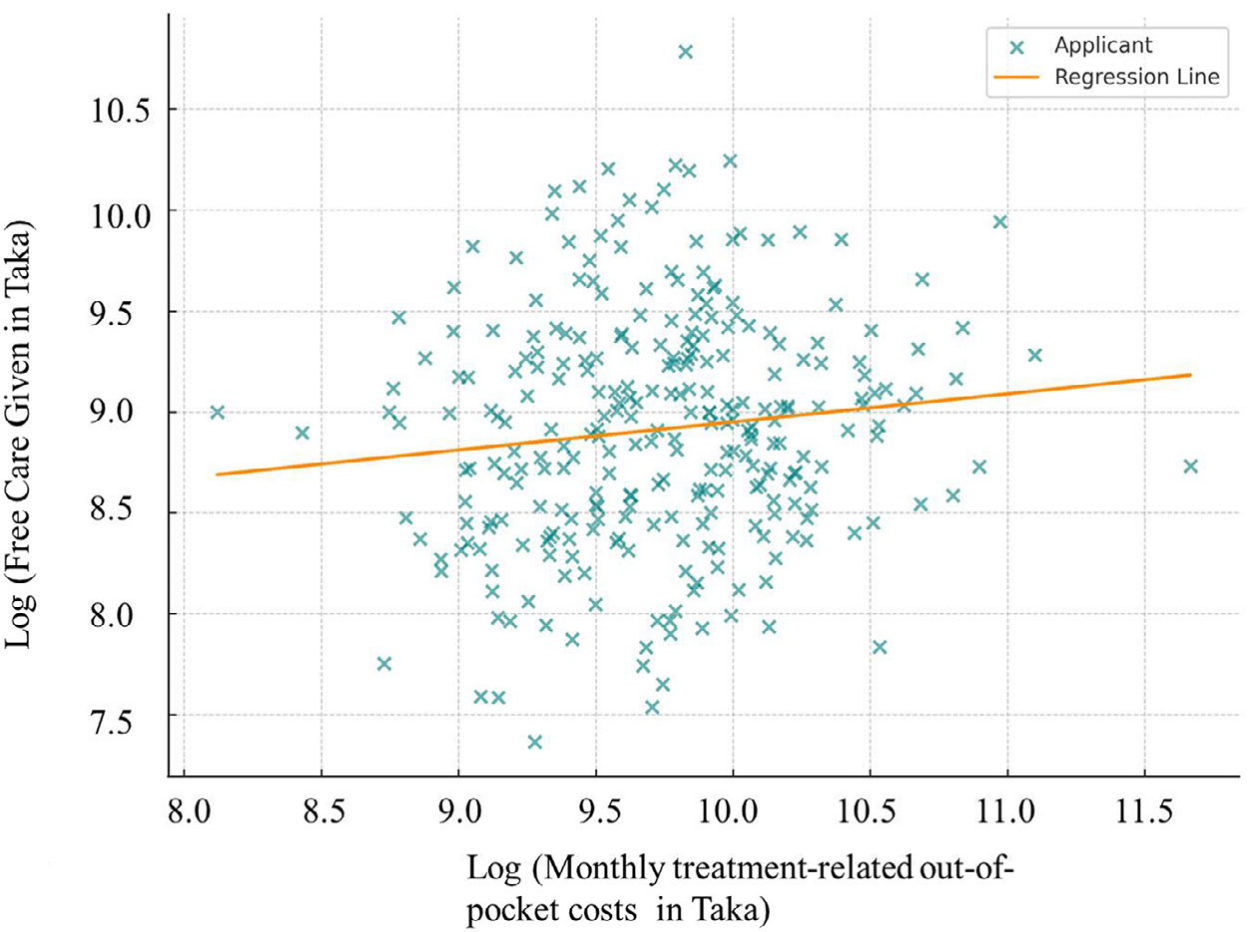
Log correlation between monthly treatment related out‐of‐pocket cost and aid (*r* = 0.168, *p* = 0.0045).

Figure 1 shows that applicants’ monthly treatment‐related OOP costs ranged broadly (mean ∼17,000 Taka), and free aid amounts averaged about 7500 Taka. The Spearman correlation between raw cost and aid was modest (*ρ* ≈ 0.138, *p* < 0.05), indicating a weak positive association. After log‐transforming both variables, Pearson's correlation was slightly stronger (*r* ≈ 0.168, *p* = 0.004), confirming that higher cost cases tended to receive more aid. This illustrates the positive correlation.

Table 2 presents the multiple regression coefficients. Controlling for all factors, monthly treatment‐related OOP cost was the strongest predictor: A one‐unit increase in log(cost) was associated with a 0.181 unit increase in log(aid) (*p* = 0.003). This indicates that each doubling of patient costs roughly increases the aid by about 18% (on average).

**TABLE 2:** Linear regression results predicting log (free care given in taka).

Predictor	Estimate (*β*)	Std. error	*p* value
Intercept	6.753	0.789	<0.001
Age: 49–69 years	0.067	0.094	0.472
Age: 7–27 years	−0.092	0.132	0.484
Age: 70–90 years	0.048	0.148	0.746
Sex: Male	−0.014	0.086	0.873
Occupation: Jobless	0.099	0.143	0.490
Occupation: Student	0.261	0.146	0.076
Marital: Married	0.284	0.223	0.206
Marital: Unmarried	0.261	0.215	0.226
Marital: Widowed	0.421	0.371	0.258
Income: Parents	−0.782	0.433	0.072
Income: Relative	−0.511	0.446	0.253
Income: Self	−0.554	0.445	0.214
Income: Spouse	−0.930	0.447	0.038
Reason: Mental health	−0.136	0.081	0.092
Reason: Other	0.014	0.106	0.897
Log(Monthly cost)	0.181	0.061	0.003

Among categorical predictors, having a spouse as the main income source was significantly associated with *lower* aid (*β* ≈ −0.930, *p* = 0.038). This negative coefficient suggests applicants with spousal financial support received about 60% less aid on average (since e^ (−0.93) ≈ 0.39) than those relying on parents (reference group), holding cost constant. Parental income was also negatively associated (*β* ≈ −0.782) but did not reach significance at the 0.05 level (*p* ≈ 0.072). Conversely, students showed a marginally positive effect (*β* ≈ 0.261, *p* ≈ 0.076), hinting that student applicants might receive slightly more aid than employed reference cases.

Reason for support had notable trends: citing mental health issues predicted a reduction in aid (*β* ≈ −0.136, *p* ≈ 0.092) compared to financial hardship. Although not statistically significant at 0.05, this suggests applicants with mental health needs received somewhat less assistance. No significant effects were found for age group, sex or marital status in the adjusted model. The overall model fit was low (adjusted *R* ^2^ ≈ 0.062), indicating that only about 6.2% of the variation in aid amount was explained by these observable factors.

## 4 Discussion

Our analysis reveals that, in this sample of aid applicants, the monetary need (proxied by monthly treatment‐related OOP costs) was the clearest driver of free‐care support. The significant positive association between patient cost and free aid suggests that disbursed funds were indeed responsive to higher expenses. This aligns with expectations that applicants with more expensive treatments represent higher financial hardship and thus elicit more help. In the literature, higher user fees or large health expenditures have been linked to increased aid or utilization when support is available [1]. For example, where user fees were abolished, the poor increased their usage of services—in our case, higher bills were effectively ‘abolished’ in part by aid [10].

The negative coefficient for having a spouse as an income source indicates that applicants from households with spousal support received significantly less aid. This makes intuitive sense that a spouse providing income likely denotes a relatively stable household resource, reducing the perceived need for external assistance. This finding echoes evidence from Ghana, where individuals losing family financial support had much lower healthcare utilization [13]. In other words, stable earnings (often via a spouse) buffer against relying on charity. Similarly, applicants supported by parents alone also trended toward less aid (*p* ≈ 0.07), suggesting that any additional household income may lead donors to allocate less. By contrast, students, who typically lack income, tended to receive more aid, highlighting that programs may consciously prioritise those without other support.

Interestingly, sex and age were not significant in the multivariate model. Although two‐thirds of applicants were female, being female did not by itself predict a different aid amount once other factors were accounted for. This contrasts with some studies (often on general healthcare use), where women utilise services more than men [8, 22]. In our context of targeted aid, gender per se did not drive differences. Likewise, older age groups did not systematically receive more or less aid after adjusting for costs; the trend was largely neutral.

The reasons for seeking aid also showed suggestive patterns. Applicants citing mental health issues received slightly less aid than those citing financial hardship, even after controlling for cost [23]. This could reflect stigma or visibility differences: Mental health problems may be less visible or elicit less empathy than acute financial crises from physical illness. Global literature underscores that stigma can deter both help‐seeking and sympathetic responses to mental illness [24]. In resource‐poor settings, NGOs and donors may prioritise tangible medical costs over psychosocial needs. This pattern warrants attention, as mental health conditions can impose heavy burdens, and equity calls for recognising these needs fully [25].

Nevertheless, our findings have practical implications. They imply that the aid program is targeting the needs that those with higher medical costs and without alternative income sources, so they do get more support. To enhance equity, program guidelines could explicitly incorporate objective criteria (e.g., income assessments) and ensure that vulnerable but less visible groups (such as those facing mental illness or women with hidden burdens) are not overlooked. Training of reviewers on social determinants may help reduce biases. Moreover, the lack of gender or marital disparities is reassuring, indicating at least no overt bias against any group.

These results are consistent with broader evidence that low socioeconomic status correlates with worse access and outcomes [7, 8]. They illustrate one facet of the financial protection goal of UHC that reducing the need for OOP payment prevents poverty and improves care‐seeking [1, 3]. Our setting is a microcosm of the free aid, which functions as a micro‐insurance. The positive correlation with cost (*r* ≈ 0.17) parallels the WHO's finding that eliminating user fees particularly benefits poorer, high‐need groups [10].

Moreover, these findings directly address the study's main goal, which is to identify socioeconomic factors that predict how need‐based mental health aid is distributed in a setting with limited resources. The results suggest that allocation decisions are not random; instead, they are significantly influenced by perceived economic vulnerability, particularly financial difficulties related to treatment and the availability of other household income. This indicates that, within the program studied, aid distribution aligns with the standard goal of financial risk protection, which is a key principle of UHC, even without formal insurance.

At the same time, the findings reveal important limitations in how need is defined and measured. Although economic considerations are frequently given precedence, other facets of vulnerability, particularly mental health‐related distress that might not manifest as overt financial hardship, seem to be of lesser importance in resource distribution. This discrepancy underscores a wider issue within mental health financing, wherein subjective need, societal stigma and inadequate mental health understanding can affect both the pursuit of assistance and institutional reactions. Consequently, the research underscores that need‐based allocation, in reality, is moulded by both objective measures and implicit social evaluations.

This study provides empirical evidence regarding the applicant characteristics linked to varying levels of financial assistance, thereby contributing to the relatively neglected field of mental health financing in low‐ and middle‐income nations. The research demonstrates that aid programs, whether charitable or NGO‐based, can effectively serve as micro‐financial protection mechanisms. However, the equitable impact of these programs is contingent upon the comprehensiveness of vulnerability definitions and assessments. Consequently, the findings broaden the scope of current debates concerning socioeconomic inequities in healthcare access by shifting the focus from service utilization alone to the allocation of financial support within mental health systems.

## 5 Limitations

The study is limited by its cross‐sectional design and reliance on administrative data. The sample is a self‐selected group of individuals who applied for aid, so findings may not generalise to all mental health service users. Cost data rely on self‐reporting, which may introduce bias. Applicants could overstate expenses to appear more needy or forget some costs. However, this bias is partially mitigated by cross‐checking reported costs against available receipts and typical local prices during data collection.

It should be noted that the adjusted *R* ^2^ of the model is relatively low (approximately 6%), indicating that a large proportion of the relative variation in aid allocation is not explained by the variables included in the model. This suggests that unmeasured factors such as clinical severity, subjective judgment by decision makers, contextual narratives, within applications, urgency of need or other administrative considerations are likely to play a substantial role in determining the amount of aid granted. Accordingly, the findings should be interpreted as identifying statistical associations rather than providing a comprehensive explanation of aid allocation decisions.

It is also important to note that several relevant socioeconomic variables, such as education level, household wealth indicators, household size, pensioner status and other markers of long‐term economic vulnerability, were not available in the administrative dataset used for this study. The absence of these variables limits our ability to fully capture the broader socioeconomic context influencing aid allocation.

## 6 Conclusion

The findings of the study reveal that free mental healthcare support distribution is largely associated with economic need. Specifically, higher OOP expenses associated with treatment are correlated with increased support. Conversely, applicants with alternative sources of household income, such as spousal or parental support, receive less aid, whereas those lacking stable income, including students, tend to receive more. To improve equity and transparency, mental health aid programs in settings with limited resources should implement more explicit, need‐based allocation frameworks that directly consider both financial hardship and mental health‐related vulnerability, thus facilitating advancements toward UHC.

## Author Contributions

Saiful Islam Saif conceptualised and designed the study. Saiful Islam Saif programmed the questionnaire and organised the data management. Sultana Nasrin and Woarisha Alam analysed the data. All authors contributed to the interpretation and discussion. Saiful Islam Saif and Sultana Nasrin drafted the manuscript. All authors read and approved the final manuscript.

## Ethics Statement

The study was reviewed by LifeSpring Consultancy Limited IRB and determined to be exempt from formal ethics approval due to use of existing, de‐identified administrative data (Reference No.2023/LS/IRB/01). The IRB reviewed the protocol and confirmed that since all personal identifiers were removed before analysis, no ethical approval was necessary.

## Consent

Written consent was obtained from participants when they enrolled in the aid program for their data to be used for program improvement. No additional research‐specific consent was needed for this retrospective analysis. Before analysis, we removed all names, phone numbers, addresses and any other identifying information. Each applicant was assigned a random study ID that cannot be traced back to them. The data were stored on password‐protected computers accessible only to the research team. We only report group results, so no individual can be identified.

## Conflicts of Interest

The authors declare no conflicts of interest.

## Data Availability

Data are not publicly available. Data are available upon request from the corresponding author.
